# Chlorido(8-hy­droxy­quinoline-κ^2^ *N*,*O*)(quinolin-8-olato-κ^2^ *N*,*O*)zinc methanol monosolvate

**DOI:** 10.1107/S160053681103337X

**Published:** 2011-08-27

**Authors:** Ezzatollah Najafi, Mostafa M. Amini, Seik Weng Ng

**Affiliations:** a Department of Chemistry, General Campus, Shahid Beheshti University, Tehran 1983963113, Iran; b Department of Chemistry, University of Malaya, 50603 Kuala Lumpur, Malaysia; c Chemistry Department, Faculty of Science, King Abdulaziz University, PO Box 80203 Jeddah, Saudi Arabia

## Abstract

In the title compound, [Zn(C_9_H_6_NO)Cl(C_9_H_7_NO)]·CH_3_OH, the Zn^II^ ion is *N*,*O*-chelated by both a neutral and a deprotonated quinolin-8-ol ligand, with a chloride ligand in the apical site completing the square-pyramidal coordination geometry. The Zn^II^ ion is displaced by 0.586 Å in the direction of the chloride ligand from the atoms forming the square plane. In the crystal, the components are linked by inter­molecular O—H⋯O hydrogen bonds, generating chains along the *b* axis.

## Related literature

For the crystal structure of 8-hy­droxy-2-methyl­quinolinium dichlorido(2-methyl­quinolin-8-olato-*κ* ^2^ *N*,*O*)zincate(II) aceto­nitrile disolvate, see: Najafi *et al.* (2011 ▶).
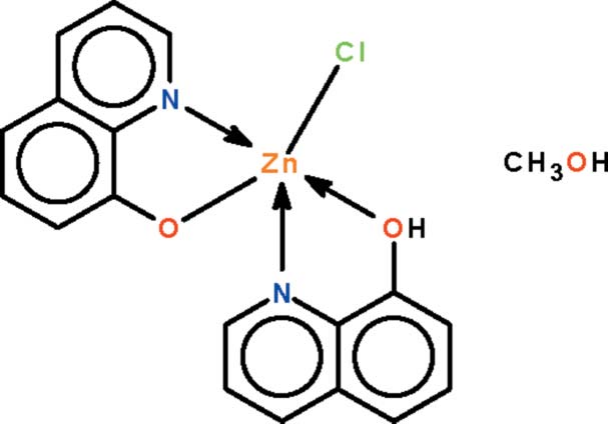


## Experimental

### 

#### Crystal data

[Zn(C_9_H_6_NO)Cl(C_9_H_7_NO)]·CH_4_O
*M* *_r_* = 422.17
Triclinic, 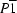

*a* = 8.4110 (4) Å
*b* = 8.4692 (4) Å
*c* = 13.2667 (7) Å
α = 99.905 (4)°
β = 95.341 (4)°
γ = 110.549 (5)°
*V* = 859.58 (7) Å^3^
*Z* = 2
Mo *K*α radiation
μ = 1.61 mm^−1^
*T* = 100 K
0.15 × 0.15 × 0.15 mm

#### Data collection

Agilent SuperNova Dual diffractometer with an Atlas detector
Absorption correction: multi-scan (*CrysAlis PRO*; Agilent, 2010 ▶) *T*_min_ = 0.795, *T*_max_ = 0.795
6878 measured reflections
3806 independent reflections
3258 reflections with *I* > 2σ(*I*)
*R*_int_ = 0.045

#### Refinement

*R*[*F* ^2^ > 2σ(*F* ^2^)] = 0.034
*wR*(*F* ^2^) = 0.082
*S* = 1.05
3806 reflections
244 parameters
2 restraints
H atoms treated by a mixture of independent and constrained refinement
Δρ_max_ = 0.47 e Å^−3^
Δρ_min_ = −0.42 e Å^−3^

### 

Data collection: *CrysAlis PRO* (Agilent, 2010 ▶); cell refinement: *CrysAlis PRO*; data reduction: *CrysAlis PRO*; program(s) used to solve structure: *SHELXS97* (Sheldrick, 2008 ▶); program(s) used to refine structure: *SHELXL97* (Sheldrick, 2008 ▶); molecular graphics: *X-SEED* (Barbour, 2001 ▶); software used to prepare material for publication: *publCIF* (Westrip, 2010 ▶).

## Supplementary Material

Crystal structure: contains datablock(s) global, I. DOI: 10.1107/S160053681103337X/lh5311sup1.cif

Structure factors: contains datablock(s) I. DOI: 10.1107/S160053681103337X/lh5311Isup2.hkl

Additional supplementary materials:  crystallographic information; 3D view; checkCIF report

## Figures and Tables

**Table 1:** Hydrogen-bond geometry (Å, °)

*D*—H⋯*A*	*D*—H	H⋯*A*	*D*⋯*A*	*D*—H⋯*A*
O2—H2⋯O3	0.84 (1)	1.71 (1)	2.547 (2)	170 (3)
O3—H3⋯O1^i^	0.84 (1)	1.76 (1)	2.592 (2)	176 (3)

Symmetry code: (i) 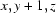.

**Fig. 1.**
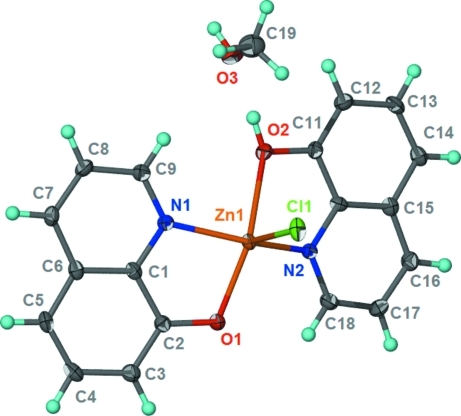
Thermal ellipsoid plot (Barbour, 2001) of ZnCl(C9H6NO)(C9H7NO)].CH3OH at the 70% probability level; hydrogen atoms are drawn as spheres of arbitrary radius.

**Fig. 2.**
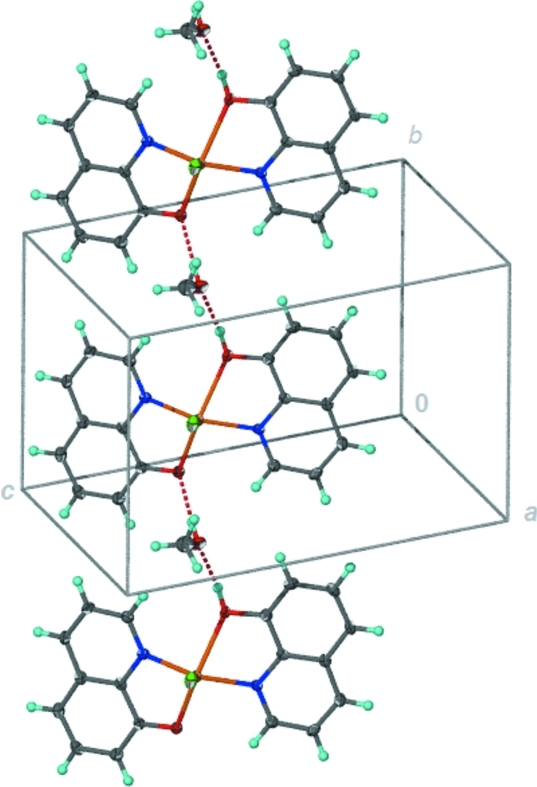
Part of the crystal structure with hydrogen bonds shown as dashed lines.

## supplementary crystallographic information

### Comment

We have previously determined the structure of 8-Hydroxy-2-methylquinolinium dichlorido(2-methylquinolin-8-olato-*κ*^2^*N*,*O*)zincate(II) acetonitrile disolvate (Najafi *et al.*, 2011). In the present study, the corresponding reaction of zinc chloride and the parent 8-hydroxyquinoline homolog in methanol yielded a neutral mono-solvated compound. The Zn^II^ atom in ZnCl(C_9_H_6_NO)(C_9_H_7_NO)]^.^CH_3_OH is *N*,*O*-chelated by a neutral as well as by a deprotonated 8-hydroxyquinoline ligand and it exists in a square-pyramidal geometry (Fig. 1). Adjacent molecules are linked by O–H···O hydrogen bonds through the solvent molecule to generate a linear chain running along the b-axis of the triclinic unit cell (Fig. 2).

### Experimental

Zinc chloride (0.13 g, 1 mmol) and 8-hydroxyquinoline (0.29 g, 2 mmol) were loaded into a convection tube and the tube was filled with methanol and kept at 333 K. Yellow crystals were collected from the side arm after several days.

### Refinement

Carbon-bound H-atoms were placed in calculated positions [C—H 0.95 to 0.98 Å, *U*_iso_(H) 1.2 to 1.5*U*_eq_(C)] and were included in the refinement in the riding model approximation.

The hydroxy H-atoms were located in a difference Fourier map, and were refined with a distance restraint O–H 0.84±0.01 Å; their temperature factors were refined.

### Crystal data

**Table:**

[Zn(C_9_H_6_NO)Cl(C_9_H_7_NO)]·CH_4_O	*Z* = 2
*M**_r_* = 422.17	*F*(000) = 432
Triclinic, *P*1	*D*_x_ = 1.631 Mg m^−^^3^
Hall symbol: -P 1	Mo *K*α radiation, λ = 0.71073 Å
*a* = 8.4110 (4) Å	Cell parameters from 3680 reflections
*b* = 8.4692 (4) Å	θ = 2.6–29.3°
*c* = 13.2667 (7) Å	µ = 1.61 mm^−^^1^
α = 99.905 (4)°	*T* = 100 K
β = 95.341 (4)°	Cuboid, yellow
γ = 110.549 (5)°	0.15 × 0.15 × 0.15 mm
*V* = 859.58 (7) Å^3^	

### Data collection

**Table:**

Agilent SuperNova Dual diffractometer with an Atlas detector	3806 independent reflections
Radiation source: SuperNova (Mo) X-ray Source	3258 reflections with *I* > 2σ(*I*)
Mirror	*R*_int_ = 0.045
Detector resolution: 10.4041 pixels mm^-1^	θ_max_ = 27.5°, θ_min_ = 2.6°
ω scans	*h* = −8→10
Absorption correction: multi-scan (*CrysAlis PRO*; Agilent, 2010)	*k* = −10→10
*T*_min_ = 0.795, *T*_max_ = 0.795	*l* = −17→17
6878 measured reflections	

### Refinement

**Table:**

Refinement on *F*^2^	Primary atom site location: structure-invariant direct methods
Least-squares matrix: full	Secondary atom site location: difference Fourier map
*R*[*F*^2^ > 2σ(*F*^2^)] = 0.034	Hydrogen site location: inferred from neighbouring sites
*wR*(*F*^2^) = 0.082	H atoms treated by a mixture of independent and constrained refinement
*S* = 1.05	*w* = 1/[σ^2^(*F*_o_^2^) + (0.0336*P*)^2^ + 0.2331*P*] where *P* = (*F*_o_^2^ + 2*F*_c_^2^)/3
3806 reflections	(Δ/σ)_max_ = 0.001
244 parameters	Δρ_max_ = 0.47 e Å^−^^3^
2 restraints	Δρ_min_ = −0.42 e Å^−^^3^

### Fractional atomic coordinates and isotropic or equivalent isotropic displacement parameters (Å^2^)

**Table:**

	*x*	*y*	*z*	*U*_iso_*/*U*_eq_	
Zn1	0.64683 (4)	0.45465 (3)	0.717891 (19)	0.01236 (9)	
Cl1	0.91659 (8)	0.57790 (8)	0.80776 (4)	0.01774 (14)	
O1	0.5263 (2)	0.2082 (2)	0.73472 (12)	0.0148 (4)	
O2	0.6496 (2)	0.6842 (2)	0.64293 (12)	0.0156 (4)	
O3	0.6795 (2)	0.9898 (2)	0.72616 (12)	0.0196 (4)	
N1	0.4816 (3)	0.5009 (2)	0.81134 (14)	0.0121 (4)	
N2	0.6744 (3)	0.3866 (2)	0.56612 (14)	0.0121 (4)	
C1	0.3948 (3)	0.3574 (3)	0.84634 (16)	0.0118 (5)	
C2	0.4213 (3)	0.2018 (3)	0.80397 (16)	0.0123 (5)	
C3	0.3344 (3)	0.0553 (3)	0.83838 (17)	0.0153 (5)	
H3A	0.3474	−0.0499	0.8109	0.018*	
C4	0.2270 (3)	0.0585 (3)	0.91326 (17)	0.0163 (5)	
H4	0.1689	−0.0450	0.9350	0.020*	
C5	0.2039 (3)	0.2065 (3)	0.95567 (17)	0.0157 (5)	
H5	0.1328	0.2064	1.0073	0.019*	
C6	0.2873 (3)	0.3594 (3)	0.92149 (17)	0.0131 (5)	
C7	0.2700 (3)	0.5192 (3)	0.95927 (17)	0.0143 (5)	
H7	0.1977	0.5269	1.0093	0.017*	
C8	0.3578 (3)	0.6616 (3)	0.92317 (17)	0.0149 (5)	
H8	0.3470	0.7689	0.9481	0.018*	
C9	0.4638 (3)	0.6488 (3)	0.84925 (17)	0.0142 (5)	
H9	0.5250	0.7490	0.8253	0.017*	
C10	0.7339 (3)	0.5164 (3)	0.51453 (16)	0.0121 (5)	
C11	0.7243 (3)	0.6787 (3)	0.55646 (17)	0.0131 (5)	
C12	0.7850 (3)	0.8125 (3)	0.50826 (18)	0.0159 (5)	
H12	0.7808	0.9216	0.5368	0.019*	
C13	0.8537 (3)	0.7903 (3)	0.41679 (18)	0.0163 (5)	
H13	0.8946	0.8849	0.3841	0.020*	
C14	0.8633 (3)	0.6356 (3)	0.37360 (17)	0.0149 (5)	
H14	0.9110	0.6233	0.3119	0.018*	
C15	0.8012 (3)	0.4946 (3)	0.42191 (17)	0.0130 (5)	
C16	0.7985 (3)	0.3275 (3)	0.38085 (17)	0.0150 (5)	
H16	0.8439	0.3067	0.3192	0.018*	
C17	0.7302 (3)	0.1966 (3)	0.43052 (17)	0.0167 (5)	
H17	0.7224	0.0829	0.4017	0.020*	
C18	0.6719 (3)	0.2314 (3)	0.52412 (17)	0.0146 (5)	
H18	0.6287	0.1402	0.5591	0.018*	
C19	0.8321 (4)	1.0712 (3)	0.8018 (2)	0.0255 (6)	
H19A	0.8904	1.1906	0.7952	0.038*	
H19B	0.9090	1.0079	0.7907	0.038*	
H19C	0.8020	1.0711	0.8714	0.038*	
H2	0.653 (4)	0.785 (2)	0.664 (2)	0.033 (9)*	
H3	0.626 (4)	1.056 (4)	0.727 (2)	0.040 (10)*	

### Atomic displacement parameters (Å^2^)

**Table:**

	*U*^11^	*U*^22^	*U*^33^	*U*^12^	*U*^13^	*U*^23^
Zn1	0.01352 (17)	0.01182 (15)	0.01295 (15)	0.00472 (12)	0.00608 (11)	0.00385 (11)
Cl1	0.0132 (3)	0.0223 (3)	0.0164 (3)	0.0050 (3)	0.0049 (2)	0.0030 (2)
O1	0.0179 (10)	0.0128 (8)	0.0163 (8)	0.0067 (7)	0.0085 (7)	0.0049 (7)
O2	0.0220 (10)	0.0138 (9)	0.0148 (8)	0.0091 (8)	0.0096 (7)	0.0039 (7)
O3	0.0210 (11)	0.0166 (9)	0.0222 (9)	0.0101 (8)	0.0017 (8)	0.0016 (7)
N1	0.0120 (11)	0.0112 (9)	0.0134 (9)	0.0046 (8)	0.0023 (8)	0.0029 (8)
N2	0.0129 (11)	0.0119 (9)	0.0136 (9)	0.0057 (9)	0.0040 (8)	0.0049 (8)
C1	0.0091 (12)	0.0139 (11)	0.0117 (10)	0.0029 (10)	0.0005 (9)	0.0046 (9)
C2	0.0127 (12)	0.0127 (11)	0.0114 (11)	0.0046 (10)	0.0023 (9)	0.0031 (9)
C3	0.0161 (13)	0.0126 (11)	0.0159 (12)	0.0042 (10)	0.0034 (10)	0.0023 (9)
C4	0.0154 (13)	0.0160 (12)	0.0156 (12)	0.0016 (10)	0.0028 (10)	0.0072 (10)
C5	0.0120 (13)	0.0191 (12)	0.0155 (11)	0.0038 (10)	0.0055 (9)	0.0049 (10)
C6	0.0092 (12)	0.0158 (12)	0.0130 (11)	0.0032 (10)	0.0015 (9)	0.0033 (9)
C7	0.0147 (13)	0.0186 (12)	0.0121 (11)	0.0079 (11)	0.0053 (9)	0.0044 (9)
C8	0.0174 (14)	0.0145 (12)	0.0158 (11)	0.0094 (11)	0.0044 (10)	0.0025 (9)
C9	0.0148 (13)	0.0136 (11)	0.0153 (11)	0.0057 (10)	0.0031 (10)	0.0048 (9)
C10	0.0125 (13)	0.0101 (11)	0.0132 (11)	0.0033 (10)	0.0015 (9)	0.0037 (9)
C11	0.0110 (12)	0.0148 (11)	0.0154 (11)	0.0058 (10)	0.0037 (9)	0.0049 (9)
C12	0.0158 (13)	0.0128 (12)	0.0193 (12)	0.0063 (10)	0.0030 (10)	0.0022 (10)
C13	0.0164 (14)	0.0152 (12)	0.0180 (12)	0.0038 (11)	0.0049 (10)	0.0089 (10)
C14	0.0153 (13)	0.0180 (12)	0.0119 (11)	0.0050 (11)	0.0042 (9)	0.0061 (9)
C15	0.0112 (12)	0.0145 (11)	0.0120 (11)	0.0039 (10)	0.0006 (9)	0.0025 (9)
C16	0.0167 (13)	0.0166 (12)	0.0118 (11)	0.0061 (11)	0.0040 (9)	0.0028 (9)
C17	0.0203 (14)	0.0120 (11)	0.0174 (12)	0.0070 (11)	0.0049 (10)	−0.0008 (9)
C18	0.0157 (13)	0.0122 (11)	0.0153 (11)	0.0038 (10)	0.0037 (9)	0.0038 (9)
C19	0.0260 (16)	0.0225 (14)	0.0262 (14)	0.0086 (13)	0.0004 (12)	0.0042 (11)

### Geometric parameters (Å, °)

**Table:**

Zn1—O1	2.0377 (16)	C7—C8	1.367 (3)
Zn1—N1	2.0392 (18)	C7—H7	0.9500
Zn1—N2	2.0547 (17)	C8—C9	1.401 (3)
Zn1—Cl1	2.2505 (7)	C8—H8	0.9500
Zn1—O2	2.3257 (16)	C9—H9	0.9500
O1—C2	1.327 (3)	C10—C15	1.410 (3)
O2—C11	1.360 (3)	C10—C11	1.424 (3)
O2—H2	0.842 (10)	C11—C12	1.363 (3)
O3—C19	1.425 (3)	C12—C13	1.404 (3)
O3—H3	0.836 (10)	C12—H12	0.9500
N1—C9	1.332 (3)	C13—C14	1.369 (3)
N1—C1	1.364 (3)	C13—H13	0.9500
N2—C18	1.328 (3)	C14—C15	1.414 (3)
N2—C10	1.366 (3)	C14—H14	0.9500
C1—C6	1.409 (3)	C15—C16	1.417 (3)
C1—C2	1.441 (3)	C16—C17	1.367 (3)
C2—C3	1.379 (3)	C16—H16	0.9500
C3—C4	1.407 (3)	C17—C18	1.399 (3)
C3—H3A	0.9500	C17—H17	0.9500
C4—C5	1.369 (3)	C18—H18	0.9500
C4—H4	0.9500	C19—H19A	0.9800
C5—C6	1.414 (3)	C19—H19B	0.9800
C5—H5	0.9500	C19—H19C	0.9800
C6—C7	1.422 (3)		
			
O1—Zn1—N1	82.18 (7)	C6—C7—H7	120.2
O1—Zn1—N2	95.64 (7)	C7—C8—C9	119.7 (2)
N1—Zn1—N2	143.92 (8)	C7—C8—H8	120.1
O1—Zn1—Cl1	112.11 (5)	C9—C8—H8	120.1
N1—Zn1—Cl1	108.81 (6)	N1—C9—C8	122.2 (2)
N2—Zn1—Cl1	105.34 (6)	N1—C9—H9	118.9
O1—Zn1—O2	150.43 (7)	C8—C9—H9	118.9
N1—Zn1—O2	90.44 (6)	N2—C10—C15	122.63 (19)
N2—Zn1—O2	73.80 (6)	N2—C10—C11	117.76 (19)
Cl1—Zn1—O2	97.40 (5)	C15—C10—C11	119.6 (2)
C2—O1—Zn1	111.33 (13)	O2—C11—C12	125.2 (2)
C11—O2—Zn1	109.79 (12)	O2—C11—C10	115.16 (19)
C11—O2—H2	109 (2)	C12—C11—C10	119.6 (2)
Zn1—O2—H2	136 (2)	C11—C12—C13	120.4 (2)
C19—O3—H3	109 (2)	C11—C12—H12	119.8
C9—N1—C1	118.9 (2)	C13—C12—H12	119.8
C9—N1—Zn1	129.73 (17)	C14—C13—C12	121.6 (2)
C1—N1—Zn1	110.92 (14)	C14—C13—H13	119.2
C18—N2—C10	118.31 (19)	C12—C13—H13	119.2
C18—N2—Zn1	122.72 (15)	C13—C14—C15	119.2 (2)
C10—N2—Zn1	117.44 (14)	C13—C14—H14	120.4
N1—C1—C6	122.56 (19)	C15—C14—H14	120.4
N1—C1—C2	116.5 (2)	C10—C15—C14	119.6 (2)
C6—C1—C2	121.0 (2)	C10—C15—C16	117.0 (2)
O1—C2—C3	124.4 (2)	C14—C15—C16	123.4 (2)
O1—C2—C1	118.4 (2)	C17—C16—C15	119.6 (2)
C3—C2—C1	117.3 (2)	C17—C16—H16	120.2
C2—C3—C4	121.4 (2)	C15—C16—H16	120.2
C2—C3—H3A	119.3	C16—C17—C18	119.5 (2)
C4—C3—H3A	119.3	C16—C17—H17	120.2
C5—C4—C3	121.8 (2)	C18—C17—H17	120.2
C5—C4—H4	119.1	N2—C18—C17	122.8 (2)
C3—C4—H4	119.1	N2—C18—H18	118.6
C4—C5—C6	119.0 (2)	C17—C18—H18	118.6
C4—C5—H5	120.5	O3—C19—H19A	109.5
C6—C5—H5	120.5	O3—C19—H19B	109.5
C1—C6—C5	119.5 (2)	H19A—C19—H19B	109.5
C1—C6—C7	116.9 (2)	O3—C19—H19C	109.5
C5—C6—C7	123.6 (2)	H19A—C19—H19C	109.5
C8—C7—C6	119.7 (2)	H19B—C19—H19C	109.5
C8—C7—H7	120.2		
			
N1—Zn1—O1—C2	−7.37 (15)	N1—C1—C6—C5	−178.8 (2)
N2—Zn1—O1—C2	−151.09 (15)	C2—C1—C6—C5	−0.1 (3)
Cl1—Zn1—O1—C2	99.82 (15)	N1—C1—C6—C7	0.9 (3)
O2—Zn1—O1—C2	−84.30 (18)	C2—C1—C6—C7	179.6 (2)
O1—Zn1—O2—C11	−92.97 (18)	C4—C5—C6—C1	−1.3 (3)
N1—Zn1—O2—C11	−167.77 (16)	C4—C5—C6—C7	179.1 (2)
N2—Zn1—O2—C11	−20.70 (15)	C1—C6—C7—C8	−0.8 (3)
Cl1—Zn1—O2—C11	83.19 (15)	C5—C6—C7—C8	178.9 (2)
O1—Zn1—N1—C9	179.1 (2)	C6—C7—C8—C9	0.1 (3)
N2—Zn1—N1—C9	−92.0 (2)	C1—N1—C9—C8	−0.5 (3)
Cl1—Zn1—N1—C9	68.3 (2)	Zn1—N1—C9—C8	−171.85 (17)
O2—Zn1—N1—C9	−29.6 (2)	C7—C8—C9—N1	0.6 (4)
O1—Zn1—N1—C1	7.27 (15)	C18—N2—C10—C15	−3.6 (3)
N2—Zn1—N1—C1	96.13 (18)	Zn1—N2—C10—C15	162.71 (18)
Cl1—Zn1—N1—C1	−103.50 (15)	C18—N2—C10—C11	175.6 (2)
O2—Zn1—N1—C1	158.53 (15)	Zn1—N2—C10—C11	−18.1 (3)
O1—Zn1—N2—C18	−22.1 (2)	Zn1—O2—C11—C12	−163.1 (2)
N1—Zn1—N2—C18	−106.6 (2)	Zn1—O2—C11—C10	18.3 (2)
Cl1—Zn1—N2—C18	92.65 (19)	N2—C10—C11—O2	−2.4 (3)
O2—Zn1—N2—C18	−173.9 (2)	C15—C10—C11—O2	176.8 (2)
O1—Zn1—N2—C10	172.22 (17)	N2—C10—C11—C12	178.9 (2)
N1—Zn1—N2—C10	87.8 (2)	C15—C10—C11—C12	−1.9 (4)
Cl1—Zn1—N2—C10	−73.00 (17)	O2—C11—C12—C13	−177.4 (2)
O2—Zn1—N2—C10	20.40 (16)	C10—C11—C12—C13	1.2 (4)
C9—N1—C1—C6	−0.3 (3)	C11—C12—C13—C14	−0.5 (4)
Zn1—N1—C1—C6	172.59 (18)	C12—C13—C14—C15	0.5 (4)
C9—N1—C1—C2	−179.0 (2)	N2—C10—C15—C14	−178.9 (2)
Zn1—N1—C1—C2	−6.1 (2)	C11—C10—C15—C14	2.0 (3)
Zn1—O1—C2—C3	−173.89 (19)	N2—C10—C15—C16	2.6 (3)
Zn1—O1—C2—C1	6.3 (3)	C11—C10—C15—C16	−176.6 (2)
N1—C1—C2—O1	−0.1 (3)	C13—C14—C15—C10	−1.3 (4)
C6—C1—C2—O1	−178.8 (2)	C13—C14—C15—C16	177.2 (2)
N1—C1—C2—C3	−179.9 (2)	C10—C15—C16—C17	0.9 (3)
C6—C1—C2—C3	1.3 (3)	C14—C15—C16—C17	−177.5 (2)
O1—C2—C3—C4	179.0 (2)	C15—C16—C17—C18	−3.2 (4)
C1—C2—C3—C4	−1.2 (3)	C10—N2—C18—C17	1.1 (4)
C2—C3—C4—C5	−0.2 (4)	Zn1—N2—C18—C17	−164.42 (19)
C3—C4—C5—C6	1.4 (4)	C16—C17—C18—N2	2.3 (4)

### Hydrogen-bond geometry (Å, °)

**Table:**

*D*—H···*A*	*D*—H	H···*A*	*D*···*A*	*D*—H···*A*
O2—H2···O3	0.84 (1)	1.71 (1)	2.547 (2)	170 (3)
O3—H3···O1^i^	0.84 (1)	1.76 (1)	2.592 (2)	176 (3)

Symmetry codes: (i) *x*, *y*+1, *z*.
